# A Randomized Phase II Trial Comparing the Efficacy and Safety of Pioglitazone, Clarithromycin and Metronomic Low-Dose Chemotherapy with Single-Agent Nivolumab Therapy in Patients with Advanced Non-small Cell Lung Cancer Treated in Second or Further Line (ModuLung)

**DOI:** 10.3389/fphar.2021.599598

**Published:** 2021-03-16

**Authors:** Daniel Heudobler, Christian Schulz, Jürgen R. Fischer, Peter Staib, Thomas Wehler, Thomas Südhoff, Thomas Schichtl, Jochen Wilke, Joachim Hahn, Florian Lüke, Martin Vogelhuber, Sebastian Klobuch, Tobias Pukrop, Wolfgang Herr, Swantje Held, Kristine Beckers, Gauthier Bouche, Albrecht Reichle

**Affiliations:** ^1^ Department of Internal Medicine III, Hematology and Oncology, University Hospital Regensburg, Regensburg, Germany; ^2^ Bavarian Center for Cancer Research, Regensburg, Germany; ^3^ Department of Internal Medicine II, University Hospital Regensburg, Regensburg, Germany; ^4^ Lungenklinik Löwenstein, Löwenstein, Germany; ^5^ Euregio Cancer Center Eschweiler, Eschweiler, Germany; ^6^ Department of Hematology, Oncology, Palliative Care, Pneumology, Evangelisches Krankenhaus Hamm, Hamm, Germany; ^7^ Lungenklinik Hemer, Hemer, Germany; ^8^ Department of Hematology and Oncology, Klinikum Passau, Passau, Germany; ^9^ Medizinisches Versorgungszentrum Weiden, Weiden in der Oberpfalz, Bavaria, Germany; ^10^ Oncology and Hematology, Fürth, Germany; ^11^ ClinAssess, Leverkusen, Germany; ^12^ Anticancer Fund, Brussels, Belgium

**Keywords:** biomodulation, anakoinosis, NSCLC, checkpoint inhibition, pioglitazone, nivolumab, priming, metronomic chemotherapy, clarithromycin

## Abstract

**Background:** Most non-small cell lung cancers occur in elderly and frequently comorbid patients. Therefore, it is necessary to evaluate the efficacy of biomodulatory active therapy regimen, concertedly interfering with tumor-associated homeostatic pathways to achieve tumor control paralleled by modest toxicity profiles.

**Patients and Methods:** The ModuLung trial is a national, multicentre, prospective, open-label, randomized phase II trial in patients with histologically confirmed stage IIIB/IV squamous (*n* = 11) and non-squamous non-small cell (*n* = 26) lung cancer who failed first-line platinum-based chemotherapy. Patients were randomly assigned on a 1:1 ratio to the biomodulatory or control group, treated with nivolumab. Patients randomized to the biomodulatory group received an all-oral therapy consisting of treosulfan 250 mg twice daily, pioglitazone 45 mg once daily, clarithromycin 250 mg twice daily, until disease progression or unacceptable toxicity.

**Results:** The study had to be closed pre-maturely due to approval of immune checkpoint inhibitors (ICi) in first-line treatment. Thirty-seven patients, available for analysis, were treated in second to forth-line. Progression-free survival (PFS) was significantly inferior for biomodulation (*N* = 20) vs. nivolumab (*N* = 17) with a median PFS (95% confidence interval) of 1.4 (1.2–2.0) months vs. 1.6 (1.4–6.2), respectively; with a hazard ratio (95% confidence interval) of 1.908 [0.962; 3.788]; *p* = 0.0483. Objective response rate was 11.8% with nivolumab vs. 5% with biomodulation, median follow-up 8.25 months. The frequency of grade 3–5 treatment related adverse events was 29% with nivolumab and 10% with biomodulation. Overall survival (OS), the secondary endpoint, was comparable in both treatment arms; biomodulation with a median OS (95% confidence interval) of 9.4 (6.0–33.0) months vs. nivolumab 6.9 (4.6–24.0), respectively; hazard ratio (95% confidence interval) of 0.733 [0.334; 1.610]; *p* = 0.4368. Seventy-five percent of patients in the biomodulation arm received rescue therapy with checkpoint inhibitors.

**Conclusions:** This trial shows that the biomodulatory therapy was inferior to nivolumab on PFS. However, the fact that OS was similar between groups gives rise to the hypothesis that the well-tolerable biomodulatory therapy may prime tumor tissues for efficacious checkpoint inhibitor therapy, even in very advanced treatment lines where poor response to ICi might be expected with increasing line of therapy.

## Introduction

Despite improvements in the first-line treatment of patients with advanced non-small cell lung cancer (NSCLC), nearly all patients experience disease progression. Management of patients with advanced NSCLC is individualized based upon molecular and histologic features of the tumor (Planchard et al., 2018). Until recently, patients with previously untreated NSCLC with no driver mutation were treated with mainly platinum-based first-line chemotherapy. Second-line treatment consisted of a different line of chemotherapy with single-agent docetaxel as the main option. The advent of inhibitors of the programmed cell death protein 1 (PD-1) or its ligand (PD-L1) in NSCLC has improved patients’ outcomes and changed the therapeutic landscape. Anti-PD-1 were first shown to be superior to docetaxel as second-line treatment (Borghaei et al., 2015; Herbst et al., 2016; Horn et al., 2017; Rittmeyer et al., 2017; Planchard et al., 2018). This was followed by results of first-line anti-PD-1 combined with chemotherapy showing superiority to chemotherapy alone in the first-line treatment of these patients (Rittmeyer et al., 2017; Gandhi et al., 2018; Socinski et al., 2018). Just when the ModuLung trial was initiated, nivolumab became a standard second-line option in both squamous and non-squamous NSCLC in Germany. However, anti-PD-1 in first line was not yet standard treatment.

Our group has shown that a combination of therapies modulating tumor angiogenesis, inflammation and immune response can result in a significant survival benefit in patients with various advanced malignancies (reviewed in (Hart et al., 2015)). This approach, called biomodulation, aims to induce communicative reprogramming of dysregulated cellular and intercellular homeostasis (anakoinosis) (Heudobler et al., 2019b). In the trial reported here, biomodulation consisted of a combination of pioglitazone, clarithromycin and low-dose metronomic treosulfan. Pioglitazone - a drug approved for type 2 diabetes—is a potent peroxisome proliferator-activated receptor (PPAR)-alpha/gamma agonist. PPAR-gamma activation has been proposed as a therapeutic strategy for NSCLC (Reichle, 2010; Giaginis et al., 2012; Khandekar et al., 2018; Ciaramella et al., 2019). Clarithromycin—a macrolide antibiotic—has strong anti-inflammatory and immunomodulatory properties, especially in the lung (Kanoh and Rubin, 2010; van Nuffel et al., 2015). A study reported that long-term treatment with clarithromycin increased the median survival of patients with non-small cell lung cancer (Mikasa et al., 1997). In cachectic NSCLC patients, clarithromycin reduced the progression of cancer-associated cachexia (Sakamoto et al., 2001). Treosulfan—an alkylating agent widely used in Germany—was used in a metronomic fashion to target the tumor microenvironment (André et al., 2014).

With limited data on biomodulation in NSCLC the ModuLung trial explores the efficacy and safety of a biomodulatory regimen compared to a standard of care (SOC) regimen; i.e., nivolumab. With previous trials in multiple entitites showing favorable safety profiles for biomodulation (Thomas et al., 2015; Vogelhuber et al., 2015; Ugocsai et al., 2016; Walter et al., 2017; Heudobler et al., 2019a) the trial also addresses the medical need for low-toxic therapies in relapsed or refractory non-small cell lung cancer (NSCLC). Moreover, with the emergence of immune checkpoint inhibitors in the NSCLC therapy landscape the study generates new hypotheses on therapy seqences and combinations as well as possible synergistic effects of immune checkpoint inhibitors and biomodulatory regimens.

## Patients and Methods

### Patients

Eligible patients were patients with histologically or cytologically confirmed locally advanced, unresectable or metastatic NSCLC who had experienced disease progression during or following treatment with a platinum-containing regimen. Patient with stage IIIB eligible for definitive chemoradiotherapy were excluded. Patients had to have an Eastern Cooperative Oncology Group performance status of 0 or 1. Patients with epidermal growth factor receptor mutation or anaplastic lymphoma kinase rearrangement were eligible if they had progressed during or after first-line targeted therapy, since further standard targeted therapy was lacking for this patient population. Patients with known active or untreated central nervous system metastases were excluded.

The institutional review boards and ethic committees of all participating centers approved the protocol (ethics committee of the University of Regensburg approval No.: 15-112-0124). The study was conducted in accordance with the Declaration of Helsinki and applicable national and European laws. All patients provided written informed consent.

### Study Design

The ModuLung trial (EUDRACT 2014-004095-31, NCT02852083) is a national, multicentre, prospective, open-label, randomized phase II trial in advanced NSCLC who failed first-line platinum-based chemotherapy. Patients were randomly assigned on a 1:1 ratio to the experimental or to the control group. Patients randomized to the experimental group received an all-oral therapy consisting of treosulfan 250 mg twice daily, pioglitazone 45 mg once daily, clarithromycin 250 mg twice daily, until disease progression or unacceptable toxicity. Dosage of each drug was choosen based on previous biomodulatory trials (Reichle A, 2010; van Nuffel et al., 2015; Vogelhuber et al., 2015; Heudobler et al., 2019a).Patients randomized to the control group received 3 mg per kilogram of nivolumab every 2 weeks until disease progression or unacceptable toxicity. No crossover was allowed between the both arms. Initially, patients randomized to the control group were to receive a docetaxel 75 mg/m^2^ intravenously on day 1 plus twice daily nintedanib 200 mg p.o. on day 2–21 (non-squamous cell histology only) of each 21-days cycle until disease progression or unacceptable toxicity, for a maximum of 6 cycles. Three patients in the control group received one or two cycles of docetaxel and nintedanib which was discontinued due to progressive disease. However, the trial protocol was rapidly amended to reflect changes in the standard treatment in the control group and all patients in the control group then received the new standard treatment (nivolumab).

Additional supportive treatment was performed according to standard practices at the participating centres. Follow-up was performed every 3 months by the local oncologist.

### Randomisation, Endpoints and Statistical Analysis

Randomisation of eligible patients was done centrally by a Contract Research Organisation (ClinAssess) and was stratified according to squamous cell or adenocarcinoma histology. The primary endpoint of the study was progression-free survival (PFS) defined as time from randomisation to progression or death from any cause, whichever occured first. Progression was defined as progressive disease according to RECIST criteria 1.1 (Eisenhauer et al., 2009). Clinical secondary endpoints were overall survival (OS), duration of response, safety, health-related quality of life using the European Organisation for Research and Treatment of Cancer (EORTC) Quality-of-Life Questionnaire Core 30 (QLQ-C30) and its Lung Cancer Module (LC13).

The study was designed to detect a change of median PFS from 3–4.5 months. Using a phase II screening design as proposed by Rubinstein and colleagues (Rubinstein et al., 2005), a power of 0.8 and an alpha of 0.20, 69 events (progression or death) were needed to show superiority of the experimental arm. To observe 69 events, 80 evaluable patients were required (40 per group). To account for an estimated drop-out rate of 5%, 86 patients were to be randomized.

The intention-to-treat population (Full analysis set) was used for all efficacy analyses. Patients' demographics and baseline characteristics were summarized using descriptive statistics (mean, standard deviation, median, minimum, and maximum values) for continuous parameters and frequencies and percentages for categorical data. PFS and OS were estimated using the Kaplan-Meier method and compared between groups by the Log-Rank test. Hazard Ratio (HR) comparing the two groups and their 95% Confidence Intervals (CIs) were estimated from a Cox proportional hazards model. Safety analyses included incidence of serious adverse events. All statistical tests were 2-sided with a 5% Type I error except for the primary endpoint for which a 20% type I error was used as per trial design. The study was powered to assess superiority of the experimental arm over the standard arm concerning PFS assuming that the standard will result in a median PFS of 3 months and that the experimental will prolong median PFS to 4.5 months.

All data were collected and analyzed using the statistical analysis software SAS^®^.

### EORTC QLQ-C30

Analysis of EORTC QLQ-C30 and the lung cancer module QLQ-C30-L13 were done according to statistical analysis plan. Nine of fifteen scales for quality of life were selected for analysis. Wilcoxon-tests were performed for comparison of the treatment arms at baseline, cycle 2, cycle 3 and final examination.

## Results

**FIGURE 1:**
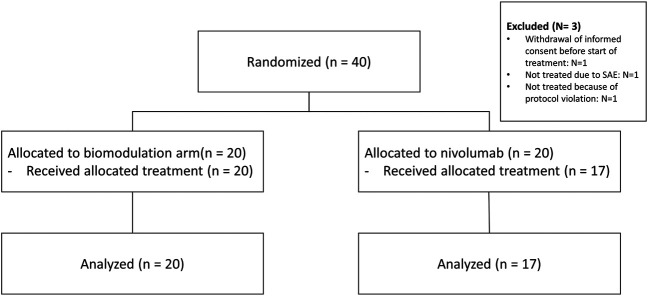
CONSORT flowchart of the ModuLung trial.

Between April 2016 and June 2018, 40 patients from seven sites in Germany were randomly assigned to the biomodulatory treatment (*n* = 20) or to nivolumab (*n* = 20). Figure 1 depicts the CONSORT diagram. The sponsor terminated the trial early because of the approval of anti-PD-1 in the first-line treatment of NSCLC. The control arm of the ModuLung trial thus became inappropriate. As there is no consensus on a standard control arm treatment following these changes, no amendment could prevent the termination of the trial. Data extract was done March 12, 2020.

### Patients’ Characteristics

Patients’ characteristics are summarized in Table 1. The CONSORT diagram (Figure 1) illustrates the repartition of the patients within the two study cohorts. Three patients were excluded due to withdrawal of consent, protocol violation and serious adverse events before receiving treatment. Therefore, the safety set comprises altogether 37 patients.

**TABLE 1:** Baseline patients’ characteristics (*N* = 37).

	Biomodulation, *N* = 20		Nivolumab, *N* = 17	
Age, years				
Mean (SD)	65.4 (±7.4)		61.2 (±7.1)	
Range	56–81		50–70	
Gender, *N* (%)				
Female	4	20%	4	24%
Male	16	80%	13	76%
Ethnicity				
Caucasian	20	100%	16	94.1%
African	0	0%	1	5.9%
ECOG performance status, *N* (%)				
0	12	60%	10	58.8%
1	8	40%	7	41.2%
Duration of disease, in months				
Mean (SD)	16.6 (±18.6)		20.5 (±20.1)	
Duration of metastatic disease, in months				
Mean (SD)	10.1 (±7.6)		13.3 (±13.2)	
Histology, *N* (%)				
Squamous cell carcinoma	6	30%	5	29%
Adenocarcinoma	14	70%	12	71%
Grading according to WHO				
G2	0	0%	1	5.9%
G3	4	20%	5	29.4%
G3-4	12	60%	8	47.1%
GX	4	20%	3	17.6%
EGFR or ALK alteration, *N* (%)				
*EGFR* wild type	11	55%	12	70.6%
* EGFR*	1	5%	0	0%
* ALK*	0	0%	1	5.9%
Unknown	8	40%	4	23.5%
Stage, *N* (%)				
IIIB	1	5%	0	0%
IVA	5	25%	5	29.4%
IVB	14	70%	12	70.6%
Location of metastatic sites				
Brain (controlled)	3	15%	0	0%
Lung, pleura	13	65%	10	58.8%
Liver	3	15%	3	17.6%
Bone	5	25%	6	35.3%
Adrenal gland	2	10%	2	11.8%
Kidney	0	0%	1	5.9%
Other	13	65%	11	64.7%
Number of metastatic sites				
1	8	40%	7	41.2%
2	8	40%	5	29.4%
3	2	10%	4	23.5%
4	1	5%	1	5.9%
5	1	5%	0	0%
Previous treatment, *N* (%)				
Platinum-based chemotherapy	20	100%	17	100%
Radiotherapy	9	45%	12	70.6%
Number of lines of chemotherapy, *N* (%)				
1	13	65%	10	59%
2	6	30%	6	35%
3	1	5%	1	6%
Consecutive therapies after progression				
Checkpoint inhibitor	15	75%	4	24%
Chemotherapy	9	45%	10	59%
No further tumor-directed therapy	3	15%	7	41%
Comorbities				
Chronic obstructive pulmonary disease	7	35%	9	53%
Coronary heart disease	8	40%	5	29%
Arrythmia	3	15%	4	24%
Occlusive peripheral arterial disease	1	5%	2	12%
Thromboemolism	4	20%	0	0%
Diabetes mellitus	5	25%	4	24%
Hypertension	6	30%	9	53%
Depression	4	20%	2	12%
Neurologic disorder	3	15%	5	29%
Renal insufficiency	2	10%	3	18%
Previous cancer	2	10%	0	0%
Smoker/former smoker	18	90%	17	100%

Patients’ characteristics were well balanced between the biomodulatory arm and the nivolumab arm, except for the number of patients with controlled cerebral metastases (3 vs. 0, respectively) and the mean time since metastatic disease (10.1 vs. 13.3 months, respectively). Within each study group similar proportions of patients were treated in second- to forth-line.

Seventeen patients (85%) in the biomodulatory arm received further anti-neoplastic therapy, 15 patients therefore (75%) received anti-PD-(L)1 (nivolumab or atezolizumab) following progression. 10 patients (59%) in the nivolumab arm were treated with further anti-neoplastic therapy following progression (Table 1).

### Efficacy

**FIGURE 2:**
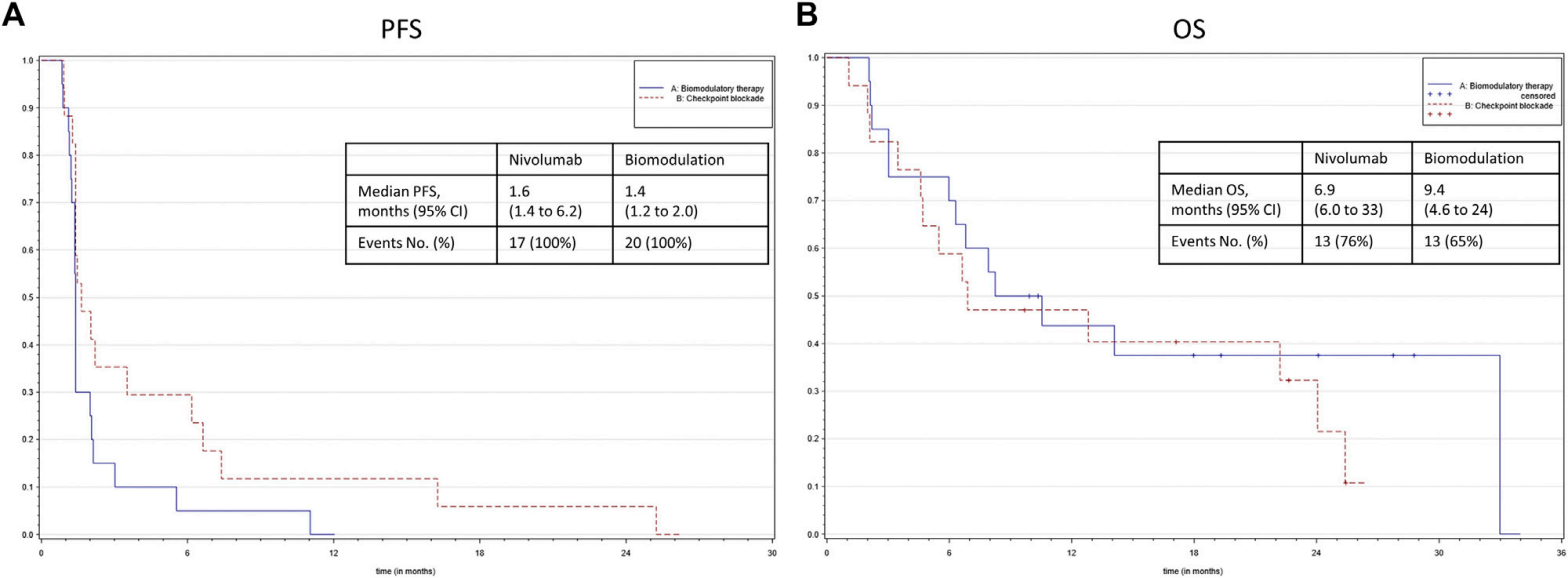
**(A)** Kaplan–Meier representation of the progression-free survival according to the treatment group. **(B)** Kaplan–Meier representation of the overall survival according to the treatment group.

After a median follow-up of 8.25 months, there was a significant difference in PFS (Figure 2) between the study arms in favor of nivolumab (HR, 1.908; 95% CI, 0.962 to 3.788; *p* = 0.0483). The median PFS time was 1.4 (1.2–2.0) months in the biomodulatory arm and 1.6 (1.4–6.2) months in the control arm B. Best response was one partial response (5%) and one stable disease (5%) in the biomodulatory arm, two partial responses (11.8%), and four stable diseases (23.5%) in the nivolumab arm; with an objective response rate of 5% vs. 11,8%, *p* = 0.584. There was no difference in the secondary endpoint median OS (HR 0.733; 95% CI 0.334–1.610; *p* = 0.4368) as shown in Figure 2, with a median OS of 9.4 (6.0–33.0) months vs. 6.9 (4.6–24.0) months, respectively.

### Safety

After a median follow-up of 8.25 months, the mean treatment duration was 2.6 months [standard deviation (SD), 3.2 months] overall, in the biomodulatory arm 2.0 months (SD, 2.4 months), in the nivolumab arm 3.4 months (SD, 3.8 months), respectively.

In the pooled safety analysis, treatment-emergent adverse events (TEAEs) and treatment-emergent serious adverse events (TESAEs) were reported in fewer patients treated with biomodulatory therapy than in patients treated with nivolumab after a median follow-up of 8.25 months. 15 patients (75.0%) in the biomodulatory arm and 14 patients (82.4%) in the nivolumab arm had at least one TEAE of any grade. Study medication-related TEAEs of any grade were observed in nine patients (45%) treated with biomodulation and in seven patients (41.2%) treated with nivolumab. Typical treatment-related select TEAEs in the biomodulation arm were peripheral edema, likely due to pioglitazone treatment, *N* = 3 (15%). Six nivolumab-treated patients had first onset of treatment-related typical AEs in GI, hepatic, renal, nervous system or pulmonary categories. There were two study medication-related TEAEs by maximum NCI-CTCAE grade 3-5 in the biomodulatory arm (10%), and five (29%) in the nivolumab arm.

The cumulative TESAEs, NCI-CTCAE grade 3–5, are presented in Table 2. No patient in the biomodulatory arm and one patient (5.9%) in nivolumab arm had at least one TESAE related to study medication. During biomodulation, NCI-CTCAE grade 3–5 toxicities leading to treatment discontinuation occurred less frequently in 5 vs. 29%, respectively. TESAEs in most categories resolved but led to death in the nivolumab arm in *N* = 3 cases (17.6%).

**TABLE 2:** Treatment-emergent serious adverse events (TESAEs) by maximum NCI-CTCAE grade 3–5 (*N* = 37)

System, organ according NCI-CTCAE		Biomodulatory therapy *N* = 20		Checkpoint blockade *N* = 17	
		N	%	N	%
Cardiac disorders	Cardiac failure	–	–	1	5.9
	Pericardial effusion	–	–	1	5.9
Gastrointestinal disorders	Autoimmune colitis	–	–	1	5.9
Infection, Infestation	Peridontitis	–	–	1	5.9
	Pneumonia	–	–	3	17.6
Injury, poisoning, procedural complications	Femur facture			1	5.9
	Thoracic vertebra fructure	1	5.0	–	–
Nervous system disorders	Cerebral hemorrhage	–	–	1	5.9
Renal and urinary disease	Renal failure	–	–	1	5.9
	Hyronephrosis	–	–	1	5.9
Respiratory, thoracic and mediastinal disorders	Pleural effusion	1	5.0	–	–
Skin and subcutaneous tissue disorders	Skin ulcer	1	5.0	–	–

In the biomodulatory arm, scheduled dose reductions were performed in 18% of the cycles for treosulfan and pioglitazone, respectively, and in 16% for clarithromycin while there were no relevant dose changes for the patients treated with nivolumab. Responders in the biomodulatory arm had also received dose reductions. Thus, there seems to be no negative impact of dose reduction on outcome.

### Quality of Life (EORTC QLQ-C30)

Quality of Life (EORTC QLQ-C30) results were available from 15 patients (75.0%) in the biomodulation arm and 12 patients (70.6%) in the nivolumab arm who filled out the questionnaire at baseline (before start of study therapy). Nine (45.0%) and 6 (35.5%) patients filled out the questionnaire at end of treatment visit. For the global health status (QL2) the *p*-values are 0.3523, 0.6094, 0.5297, and >0.9999. As well, no statistical significant differences between the treatment arms were detectable for the other eight scales.

## Discussion

Our trial, though terminated prematurely, shows that biomodulatory treatment is inferior to nivolumab on PFS. There was no difference in OS with the limitation that the study was not powered to assess non-inferiority/superiority concerning the secondary endpoint OS. Quality of life was similar in both arms, but differences in terms of toxicity were observed. With a proportion of more than one third of patients enrolled for third- and fourth-line in both study arms, PFS for the control arm, nivolumab, is expectedly inferior to that in the CheckMate 017 and CheckMate 057 trials or atezolizumab treatment (Horn et al., 2017; Rittmeyer et al., 2017). The result is in line with retrospective data on decreasing efficacy of nivolumab with increasing number of preceding lines of chemotherapy (Rizvi et al., 2015; Lang et al., 2019).

PFS in the biomodulatory treatment arm is significant inferior to nivolumab treatment. Nevertheless, biomodulation with pioglitazone, clarithromycin and metronomic treosulfan emphasizes for the first time that the combination has immediate concerted activity resulting in objective response and disease stabilization. Surprisingly preceding biomodulation might have reached out on outcome of consecutive third- to fifth-line therapy with nivolumab, as indicated by a median OS rate in the experimental arm comparing with that of second-line treatment in CheckMate 017 and CheckMate 057 trial (Horn et al., 2017).

The clinical discrepancy between poor PFS in the biomodulation arm and a corresponding median OS equal to nivolumab second-line therapy may be discussed on the background of pre-clinical data showing multifold biomodulatory activities of the drug components administered in the experimental schedule (Teresi et al., 2006, 2006; Gottfried et al., 2011; Ge et al., 2012; Peng et al., 2015; van Nuffel et al., 2015; Heudobler et al., 2018b; Chowdhury et al., 2018; Heudobler et al., 2019b; Renner et al., 2019; Bahrambeigi et al., 2020; Batyrova et al., 2020; Fu et al., 2020). Summarizing the present clinical results and preclinical findings on the activity profile of single scheduled drugs, both datasets are hypothesis generating and suggest an impact of biomodulation on efficacy of the consecutive nivolumab therapy in third to fifth line. That means, biomodulation targeting dysregulated tumor-associated homeostatic pathways, not only induces objective response and disease stabilization, but also provides the prerequisites in tumor tissue for improving clinical activity of checkpoint inhibitor therapy as indicated by a similar OS compared with the standard arm.

### Contextualization

Currently, two questions in context of the treatment with checkpoint inhibitors still remain unanswered: First, what are the best combination partners for checkpoint inhibition, not only showing additive activity, like cytotoxic drugs (Chen et al., 2019)? Second, how can we prevent or break the frequently observed resistance to checkpoint inhibitors to establish successful re-challenge of checkpoint inhibitor therapy (Giaj Levra et al., 2020; Metro and Signorelli, 2020)?

The study results may suggest that the chosen biomodulatory therapy preceding the frequently used nivolumab/atezolizumab rescue treatment may enhance checkpoint inhibitors’ activity profile in advanced therapy line. Even if retrospectively collected data on NSCLC without driver mutations indicate that nivolumab is working continuously worse with increasing number of preceding chemotherapies, the OS rate for biomodulation in the current trial compares with those of second-line treatment in Checkmate studies (Lang et al., 2019).

The activity profiles of the single biomodulatory components, combined in the current treatment schedule, support the hypothesis of a priming effect on tumor tissue. ‘Educating’ tumor tissue seems to be possible, although the single components have poor or no monoactivity.

According to the literature, peroxisome proliferator-activated receptors (PPAR)α/γ agonists may increase tumor suppressor expression, as shown for phosphatase and tensin homolog (PTEN) (Teresi et al., 2006). Enhancing the expression of none-mutated tumor suppressor genes, decisively attenuates tumor proliferation (Teresi et al., 2006). Upregulation of deregulated tumor suppressor genes in T-lymphocytes may particularly enhance their motility, a conditio *sine qua non* for sufficient activity of checkpoint inhibitors (Peng et al., 2015). Further, PPARα/γ agonists regulate tumor cell metabolism, proliferation and down-regulate inflammation (Gottfried et al., 2011). Moreover, T-cell activity may be modulated by regulating metabolic processes in T-lymphocytes, such as inhibition of glycolysis via the PPARα agonistic component (Chowdhury et al., 2018; Renner et al., 2019). Restricting glycolysis preserves T cell effector functions and augments checkpoint therapies (Renner et al., 2019). Activation of the PGC-1α/PPAR pathway during PD-1 blockade reprograms the effector T-cell metabolism, retains T-cell proliferative capacity and prolongs T-cell survival.

PPARα/γ agonists may even enhance PD-1 expression (Chowdhury et al., 2018; Renner et al., 2019). Additionally, PPARα/γ agonists modulate the impaired lipid biosynthesis, which hinders anti-tumor efficacy of intratumoral natural killer T-cells (iNKT) (Fu et al., 2020).

Metronomic chemotherapy may give rise to the production of tumor-specific T-cells, besides anti-angiogenic effects (Ge et al., 2012). Clarithromycin also exerts pleiotropic effects, acts immunomodulatory and angiostatic, as also shown for NSCLC (van Nuffel et al., 2015).

Thus, the drug cocktail of the biomodulatory treatment arm may contribute to correcting aberrant homeostasis in cancer tissue, beyond the expected main effect, namely apoptosis induction, a procedure called anakoinosis (Heudobler et al., 2019b).

A clinically, still underestimated phenomenon following chemotherapy or classic targeted therapy is the regularly observable therapy-induced host cells’ functional shift (Shaked, 2019), or resistance constituted by non-selective mechanisms, e.g. metabolic plasticity of tumor tissues (Desbats et al., 2020). In contrast to classic targeted therapies and cytotoxic therapies, novel anakoinosis-inducing therapies may meet potential challenges arising with treatment-associated secondary changes in tumor tissue by establishing novel tissue homeostasis in a therapeutically meaningful way (Heudobler et al., 2019b). A time to second objective disease progression (PFS2) benefit in prospective trials or the induction of ‘biological memory’ clearly indicate the sustainable biomodulatory activity profile of drugs or drug combinations acting as pro-anakoinotic ‘master modifiers’ of tumor tissue (Hart et al., 2015; Jackson et al., 2019; Bahlis et al., 2020). ‘Master modifiers’ may prime tumor tissue in a clinically relevant way (Hart et al., 2015; Heudobler et al., 2018a; Heudobler et al., 2019b). The major novel therapeutic aspect introduced by anakoinosis is considering tumor disease as a non-cell autonomous disease also in terms of therapeutic purposes (Heudobler et al., 2019b).

Our study results may allow to substantiate the mentioned hypothesis, especially in context with multifold hypothesis supporting pre-clinical data, that biomodulation facilitates reprogramming of tumor tissue’s homeostasis, thereby priming NSCLC without driver mutations for more efficacious checkpoint inhibitor therapy or even breaking PD-1/PDL1 resistance. Therefore based on the preclinical data as well as the results of the ModuLung trial it seems very promising to explore combinations of biomodulatory and immune checkpoint inhibitor therapy in future clinical trials in NSCLC.

### Strengths and Limitations

The approval of checkpoint inhibitors as first line therapy for NSCLC without driver mutations led to premature termination of the study. Consecutively, the limited number of patients in each treatment arm did not allow to compensate differences in patients’ characteristics, e.g. controlled brain metastases in the experimental arm and a shorter time from initial diagnosis to treatment start (Martin et al., 2020; Youn et al., 2020). Although, PFS was statistically significantly different between the study groups, the secondary endpoint, median OS, gains particular importance, as the frequently applied consecutive checkpoint inhibitor therapy in patients treated in the experimental arm completely equalized presumable disadvantages of a preceding biomodulatory therapy or imbalanced patient characteristics. In this context, it has to be stressed that the study was not powered to assess non-inferiority concerning the secondary endpoint OS. Moreover unfortunately, data on PFS2 are not available.

In summary, subsequent to this ostensibly negative result for biomodulatory therapy concerning the primary endpoint PFS, the biomodulatory therapy induces response in advanced NSCLC, being translated in equivalent OS, compared to the standard arm. Moreover, the beneficial median OS based on successful consecutive checkpoint inhibitor therapy (65% patients received nivolumab in third- to fifth-line), gives rise to the hypothesis that the administered biomodulatory therapy primes tumor tissue for efficacious checkpoint inhibitor therapy, even in very advanced treatment lines. This hypothesis should be tested in subsequent trials assessing the efficacy and safety of the promising combination of biomodulatory and immune checkpoint treatments. Biomodulation in NSCLC had a well tolerable safety profile. Tolerability of treatment schedules in advanced treatment lines is particularly important, as multivariate analysis in patients receiving nivolumab reveals a significantly increased hazard of death, in case of multiple comorbid conditions (Martin et al., 2020; Youn et al., 2020).

## Data Availability

The raw data supporting the conclusions of this article will be made available by the authors, without undue reservation.
